# Equitable Timing of HIV Diagnosis Prior to Pregnancy: A Canadian Perspective

**DOI:** 10.7759/cureus.16691

**Published:** 2021-07-28

**Authors:** Esther S Shoemaker, Kate Volpini, Stephanie Smith, Mona Loutfy, Claire Kendall

**Affiliations:** 1 Internal Medicine, C.T. Lamont Primary Health Care Research Centre, Bruyère Research Institute, Ottawa, CAN; 2 Internal Medicine, Institute for Clinical Evaluative Sciences (ICES), Toronto, CAN; 3 Internal Medicine, Ottawa Hospital Research Institute, Ottawa, CAN; 4 Internal Medicine, University of Ottawa, Ottawa, CAN; 5 Medicine, C.T. Lamont Primary Health Care Research Centre, Bruyère Research Institute, Ottawa, CAN; 6 Infectious Disease, Women’s College Research Institute, Women’s College Hospital, Toronto, CAN; 7 Internal Medicine, University of Toronto, Toronto, CAN; 8 Family Medicine, C.T. Lamont Primary Health Care Research Centre, Bruyère Research Institute, Ottawa, CAN; 9 Family Medicine, Institute for Clinical Evaluative Sciences (ICES), Toronto, CAN; 10 Family Medicine, Ottawa Hospital Research Institute, Ottawa, CAN; 11 Family Medicine, University of Ottawa, Ottawa, CAN; 12 Family Medicine, Li Ka Shing Knowledge Institute, St. Michael’s Hospital, Toronto, CAN

**Keywords:** family planning, hiv, infectious disease, obstetrics, preconception care, screening, family planning

## Abstract

Initiating antiretrovirals prior to conception leads to a negligible risk of perinatal transmission. This study aimed to determine the timing of HIV diagnosis among pregnant women with HIV in Ontario. A retrospective population-level cohort study using linked health administrative databases was conducted to establish maternal HIV status and timing of HIV diagnosis of all women living with HIV who gave birth in 2006-2018. The majority of the 1012 women living with HIV who gave birth in Ontario were diagnosed prior to pregnancy (87.9%); however, many were not (12.1%). Among those diagnosed during pregnancy, only 23% were diagnosed in the first trimester. While HIV screening tests are being well directed towards young women, several women still enter pregnancy undiagnosed and are not diagnosed early. This calls for a continuous effort to promote universal pre-conception screening and to use HIV point-of-care testing for at-risk pregnant women and those presenting late to prenatal care.

## Introduction

Since 1999, Ontario, the Canadian province with the largest population of people living with HIV [1], has offered universal HIV screening to all pregnant women, leading to a 61.9% increase in prenatal HIV testing from 1999 to 2009 [2]. Nonetheless, 21% of HIV cases remain undiagnosed in the general population [3], lending itself to the possibility that a proportion of women living with HIV are unaware of their HIV status prior to conception or initial prenatal screening. This high burden of undiagnosed HIV is comparable to estimates within European countries [4] and the United States [5].

The timing of HIV diagnosis in relation to pregnancy is important for the care and health outcomes of mothers and infants. When proper anti-retroviral therapy is initiated prior to conception or very early during pregnancy and adhered to during pregnancy, viral suppression can be achieved resulting in virtually no perinatal HIV transmission [6]. Similarly, the earlier anti-retroviral therapy is initiated during pregnancy in treatment-naïve women, the increased likelihood that viral load is negligible by the time of delivery and that the risk of perinatal transmission is minimized [7]. Commencing anti-retroviral therapy prior to pregnancy also allows for potential drug-related side effects to be managed before they become exacerbated by symptoms of pregnancy [8]. Current obstetrical guidelines by the Society of Obstetricians and Gynecologists of Canada recommend all women living with HIV who are trying to conceive initiate anti-retroviral therapy immediately in the pre-conception period [9]. Pregnant women living with HIV are also at increased risk for active hepatitis B, anogenital group B Streptococcus (GBS) colonization, vulvovaginal candidiasis, and sexually transmitted infections (i.e., herpes simplex virus (HSV), syphilis, chlamydia) [10]. Many of these infections can negatively impact birth outcomes and newborn health, further necessitating the need for appropriate counselling and screening before initiating pregnancy. However, this can only be achieved if the identified risk factors are known to the physician and patient in advance. In addition, a new diagnosis of HIV is a significant life event that often requires additional medical appointments and counselling and that can be associated with heightened feelings of anxiety and depression [11]. The balancing of multiple health care appointments during pregnancy (i.e., infectious disease, obstetrics, and mental health) can be both time-consuming and exhausting, especially for women who already face barriers that increase their risk for poor retention in care. Combating the mental health challenges associated with a new HIV diagnosis, while adjusting to pregnancy, can further exacerbate the stress experienced by these women [12].

Despite the clear advantages of identifying and diagnosing women with HIV prior to their pregnancies, recent studies out of the United States of America have revealed that as many as 25%-32% of women do not learn of their positive HIV-status until post-conception [13,14]. Given the importance of early HIV diagnosis and considering differences between American and Canadian healthcare systems, notably differences in statutes for prenatal HIV testing [15,16] and accessibility to physicians and laboratory services [17], our objective was to investigate if these trends in the timing of HIV diagnosis are comparable. We set out to determine how Ontario is performing at identifying women living with HIV prior to them becoming pregnant. We further aimed to distinguish the percentage of pregnancy-related HIV diagnoses into those made in the first, second, and third trimester of pregnancy.

## Materials and methods

Health administrative databases were accessed using unique encoded identifiers at ICES, formally known as the Institute for Clinical and Evaluative Sciences, to link Ontario women who gave birth between April 1, 2006 and March 31, 2018 to the Ontario HIV database to establish maternal HIV status and timing of HIV diagnosis [18]. Women living with HIV were defined as having at least three Ontario Health Insurance Plan (OHIP) physician claims for HIV diagnosis within a three-year period. Women were identified using the MOMBABY database, including inpatient admission records for >98% of all in-hospital births [19]. We further assessed the following demographic variables. Immigration data were accessed through the linked Immigration, Refugee and Citizenship Canada Permanent Resident Database (IRCC-PRD) and immigration status was categorized into recent immigrants (having arrived equal or less than five years prior), long-term immigrants (having arrived greater than five years prior), and long-term residents of Canada (having no record in the immigration database). Region of origin (Canada or other versus African or Caribbean) was determined on the landing date in Ontario. We applied the rurality Index for Ontario (RIO) scoring tool to define communities as urban (RIO 0-39) or rural (RIO 40+) [20]. To evaluate income quintile, we correlated postal codes with data from the Statistics Canada census to estimate neighbourhood income ranging from 1-lowest to 5-highest. Mental health comorbidity was defined as having at least two mental health service claims in the OHIP database. We used ICD10-CA mental health diagnostic codes corresponding to the Diagnostic and Statistical Manual of Mental Disorders 4th and 5th edition for schizophrenia and psychotic disorders, non-psychotic disorders, mood and anxiety disorders, and substance use disorders. We used descriptive statistics to summarize the demographic characteristics of women living with HIV who have given birth in Ontario since 2006/07 and the timing of their HIV diagnosis stratified by three-year intervals (2006/7-2009/10, 2010/11-2013/14, and 2014/15-2017/18).

Our research team includes women with lived experience following the Community Scholar model [21], which is consistent with the Greater Involvement and Meaningful Engagement of People and Women Living with HIV/AIDS (GIPA/MEPA/GIWA/MEWA) principles [22], with the Public Health Agenda of Canada (PHAC)’s Framework to Reduce the Public Health Impact of Sexually Transmitted and Blood-Borne Infections in Canada by 2030, and CIHR’s Strategy for Patient-Oriented Research Capacity Building and Patient Engagement Frameworks [23]. The Community Scholars contributed to setting the research agenda, determining the research question, interpreting the data, and sharing the results.

## Results

During the 12-year study period, a total of 1012 women living with HIV gave birth in Ontario. The mean age of women living with HIV at the time of giving birth was 32.7. Within our sample, 97.8% of women lived in an urban setting and 47.1% fell within the lowest income quintile. Additionally, 44.7% of women identified as African or Caribbean, compared to 55.3% of women identifying their region of origin as Canadian or other. When focusing on immigration status, we found that among the women in our total sample, 20.8% were recent immigrants, 29.5% were long-term immigrants, and 47.2% were either Canadian or had landed outside of Ontario. Lastly, when examining the prevalence of mental health comorbidities, we found that only 21.3% of women living with HIV were receiving mental healthcare, while 78.7% of women were not. There were no significant differences in baseline characteristics across years. Detailed demographic characteristics by year interval are provided in Table 1.

**Figure 1:**
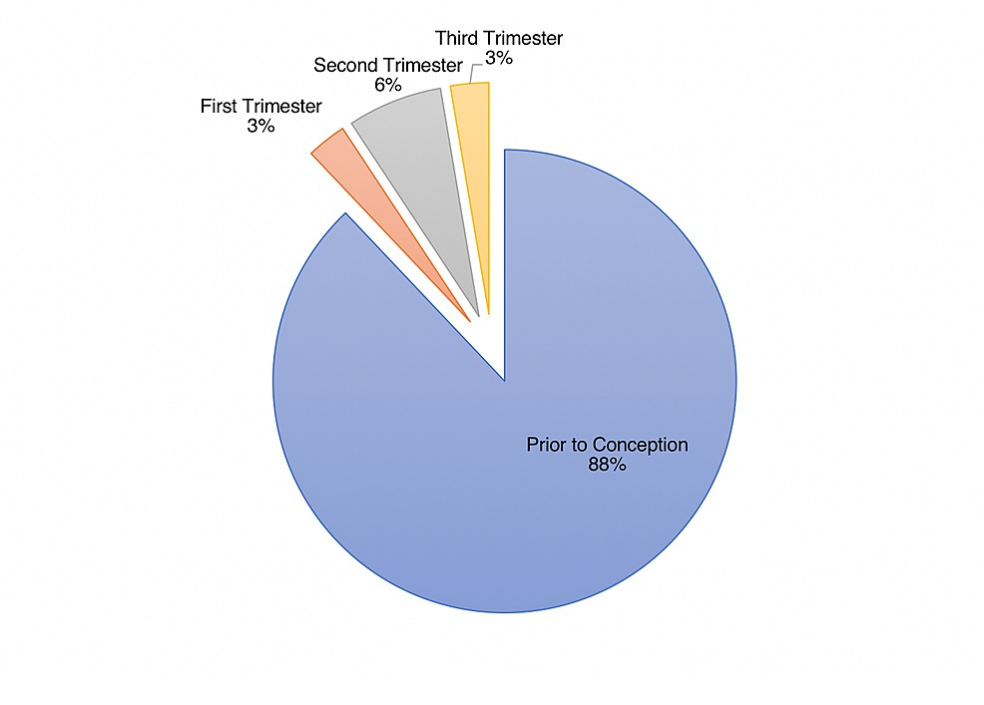
Timing of HIV diagnosis among pregnant women living with HIV between 2006-2018

 Within our cohort, 87.9% of women were diagnosed with HIV prior to conception. Among those diagnosed during pregnancy, 23% were diagnosed in the first trimester, 54.9% in the second trimester, and 22.1% in the third trimester, which was consistent across year intervals (Figure 1). When stratified into three-year periods, the longitudinal data suggests an initial decreasing trend in the proportion of HIV diagnoses during pregnancy, with 15.1% in the 2006/07-2009/10 cohort and 10% in the 2010/11-2013/14 cohort. This rate has since remained relatively stable (Table 2).

**Table 1:** Demographic characteristics of women living with HIV who have given birth in Ontario *Cell sizes <6 are not reported to ensure privacy

Characteristics	2006/07 - 2009/10 n=312 n (%)	2010/11 - 2013/14 n=360-370 n (%)	2014/15 - 2017/18 n=326-336 n (%)	TOTAL n=1012 n (%)
Age (mean, SD)	31.00 ± 5.26	32.70 ± 5.46	33.26 ± 5.98	32.36 ± 5.65
Immigrant Status				
Canadian or landed outside of Ontario	149 (47.8)	167 (45.3)	162 (48.9)	478 (47.2)
Recent immigrant (<5 years)	73 (23.4)	72-77	57-61	210 (20.8)
Long-term immigrant (≥5 years)	77 (24.7)	111 (30.1)	107 (32.3)	295 (29.2)
Missing	13 (4.2)	10-15	1-6	29 (2.8)
Region of Origin				
Canadian or other	169 (54.2)	195-200	190-195	560 (55.3)
African/Caribbean	143 (45.8)	165-170	136-141	452 (44.7)
Rurality				
Rural	<=5	1-5	<=5	16 (1.6)
Urban	305-310	355-360	321-326	990 (97.8)
Missing	<=5	<=5	<=5	6 (0.6)
Income Quintile				
1 (lowest)	153 (49.0)	171 (46.3)	153 (46.2)	477 (47.1)
2	58 (18.6)	75 (20.3)	64 (19.3)	197 (19.5)
3	37 (11.9)	55-60	52-57	148 (14.6)
4	41 (13.1)	34-39	33-38	113 (11.2)
5 (highest)	20 (6.4)	25 (6.8)	24 (7.3)	69 (6.8)
Any Mental Health Comorbidity				
Yes	62 (19.9)	78-83	70-75	216 (21.3)
No	250 (80.1)	282-287	256-261	796 (78.7)

**Table 2:** Timing of HIV diagnosis among pregnant women living with HIV *Cell sizes <6 are not reported to ensure privacy

Timing of diagnosis	2006/07 - 2009/10 n=312 n (%)	2010/11 - 2013/14 n=360-370 n (%)	2014/15 - 2017/18 n=326-336 n (%)	TOTAL n=1012 n (%)
HIV diagnosed prior to conception	265 (84.9)	332 (90.0)	293 (88.5)	890 (87.9)
HIV diagnosed during first trimester	12 (3.8)	1-6	10-15	28 (2.8)
HIV diagnosed during second trimester	23 (7.4)	26 (7.0)	18 (5.4)	67 (6.6)
HIV diagnosed during third trimester	12 (3.8)	1-6	5-10	27 (2.7)

## Discussion

These results highlight Ontario’s success in identifying women living with HIV, underlying the continuous progress within Canada towards the elimination of perinatal HIV transmission. Compared to the United States, a significantly greater proportion of Ontario women are tested for HIV prior to pregnancy, likely due to universal access to physician and laboratory services [14]. It is important to note that our study finding of 88.5% of women living with HIV receiving their HIV diagnosis prior to conception exceeds the estimated 65% proportion of the general Ontario population who were aware of their positive HIV status in 2010 [24]. While our data does not allow us to assess the correlation between awareness of HIV status and engagement in HIV care or antiretroviral therapy, we suggest that women who know their HIV status have a higher probability of being already connected to resources and an HIV care management team, prior to visiting a prenatal care provider. We believe this to be reassuring to prenatal care providers who might otherwise be worried about the additional pressures and responsibilities in managing a new diagnosis, like HIV, during pregnancy. We are encouraged that our data aligns with the first stage of the UNAIDS “90-90-90” targets for diagnosis, treatment, and viral suppression [25], and we support future global health initiatives aimed at investigating the proportion of pregnant women living with HIV receiving antiretroviral therapy and achieving viral suppression during pregnancy.

In Canada, as found globally, women of reproductive age represent one of the growing demographics of people living with HIV, and women living with HIV have both increased desires for pregnancy and are increasingly becoming pregnant [26]. Early diagnosis and treatment of HIV may help establish confidence, in women and providers, that healthy antenatal outcomes can be achieved. While our data does not allow us to assess the correlation between awareness of HIV status and engagement in HIV care or antiretroviral therapy, we suggest that women who know their HIV status have a higher probability of being already connected to resources and an HIV care management team, prior to their first visit with a prenatal care provider. We consider this to be reassuring to clinicians who might otherwise be worried about the additional pressures and responsibilities in managing a new diagnosis, like HIV, during pregnancy.

We also identified that a significant proportion of women living with HIV remained undiagnosed until pregnancy with higher rates of diagnosis occurring during the second trimester, compared to during the first and third trimesters. We suggest this trend relates to delays to initiate prenatal bloodwork and the time it takes to receive the results for prenatal serologic tests. Women living with HIV and women at-risk for HIV often experience marginalization and barriers that prevent them from accessing preconception counselling and routine prenatal care. Only 50.8% of women living with HIV initiate prenatal care in the first trimester, compared to 70% of other women [27]. While these findings relate to women with known HIV status, we argue that they likely extend to women at risk for acquiring HIV as well. We emphasize the importance of promoting universal pre-conception screening rather than relying on prenatal HIV testing, which is unavailable until pregnancy is well established. To expedite HIV diagnosis in pregnancy, we advocate for increased uptake of HIV point-of-care testing in at-risk populations and those presenting late to prenatal care.

An additional possibility for not having been diagnosed with HIV prior to conception is that women did not actually contract HIV until the time of conception or early during their pregnancy. In a recent Italian study, 15% of women with perinatal transmission to child tested negative at the start of pregnancy and developed an acute infection during pregnancy [28]. Future studies should explore whether women diagnosed during the third trimester had previously tested negative.

## Conclusions

Our study exemplifies that many women of reproductive age in Ontario have access to HIV testing and we hope to inspire other international healthcare communities that such a target is achievable. While we expected the overall proportion of women diagnosed during pregnancy to be even higher given the proportion of people undiagnosed in the general population, as well as the increasing rate of women living with HIV, our data instead showed that within Ontario, HIV screening tests are being well directed towards women of reproductive age. With early HIV diagnosis, antepartum care can better target the pregnancy-related needs of women living with HIV (i.e. monitoring of viral loads, screening for comorbidities, mental health counselling). The availability of efficacious HIV treatments during pregnancy, especially if started early, justifies the continuous push to identify all women living with HIV who are unaware of their status.
